# Ocular pathology of uncommon hematologic malignancies: a case series

**DOI:** 10.1186/1752-1947-1-158

**Published:** 2007-11-28

**Authors:** James E Head, Defen Shen, Maribel Santiago-Maysonet, Rachel J Bishop, Chi-Chao Chan

**Affiliations:** 1 Immunopathology Section, National Institutes of Health, Bethesda, MD, USA; 2 Clinical Research Training Program, NIH, Bethesda, MD, USA; 3 Consult Services Section National Eye Institute, National Institutes of Health, Bethesda, MD, USA

## Abstract

**Introduction:**

In general, ocular complications of hematologic malignancies such as leukemia are well documented. However, reports of ocular involvement in such diseases as lymphomatoid granulomatosis and chronic myelomonocytic leukemia are uncommon. Here we present cases of these two relatively rare hematologic malignancies demonstrating clinical and subclinical ocular involvement.

**Case Presentation:**

In the first case, a 54-year-old man with a previous diagnosis of lymphomatoid granulomatosis presented with a new-onset conjunctival lesion while his systemic disease was thought to be in remission. A biopsy was taken that revealed heavy infiltrates of B and T cells at the site of the lesion. Molecular analysis confirmed that these cells were positive for both Epstein-Barr viral DNA and immunoglobulin heavy chain gene rearrangement, consistent with a manifestation of his systemic disease. In the second case, a 51-year-old man with chronic myelomonocytic leukemia died after a waxing and waning clinical course. Post-mortem studies revealed the presence of atypical monocytes in the choroidal and subretinal spaces, consistent with his previous diagnosis.

**Conclusion:**

While ocular involvement in hematologic malignancies is not uncommon, these two cases describe involvement of the eye by two relatively rare neoplasms. We herein emphasize novel findings in each case, including conjunctival involvement as the first sign of recurrent lymphomatoid granulomatosis and the combination of subretinal and choroidal myelomonocytic leukemic infiltration. With the evolution of new antineoplastic therapies that may prolong life, these cases exemplify the importance of eye care in patients diagnosed with hematologic malignancies.

## Introduction

Ocular complications of hematologic malignancies such as leukemia are well documented. It is estimated that 50% or more of all leukemias manifest some form of ocular involvement [1,2]. Here we present the cases of two patients with relatively rare hematologic malignancies with notable pre- or post-mortem demonstrating clinical and pathological ocular involvement.

## Case presentation

### Case 1

**Figure 1:**
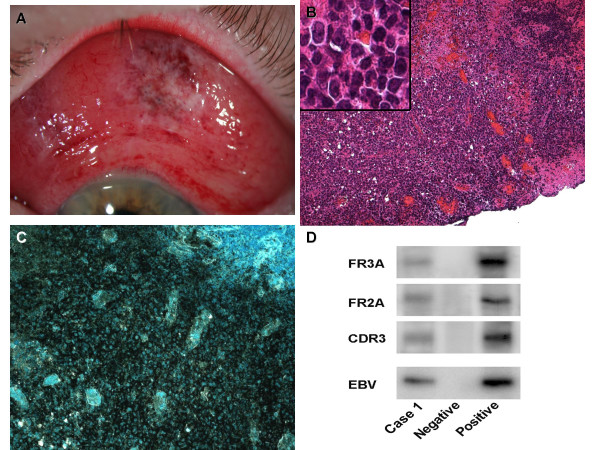
*Case 1*. (A) Generalized conjunctival injection, chemosis and a prominent lesion with elevated, polygonal, hyperemic mounds and small hemorrhages are seen in the superior bulbar conjunctiva. (B) A dense infiltration of cells and several foci of necrosis are present, as is hemorrhage into the conjunctival parenchyma. The inset demonstrates pleomorphism and prominence of nucleoli. (C) Immunohistochemistry demonstrates strongly immunopositive staining for B-cell marker, CD20. (D) Gel electrophoresis reveals: (lane 1) positive bands are indicative of IgH gene rearrangements for B-cell lymphoma. EBV DNA is detected in the lymphoma cells. (Lane 3 = negative control, lane 4 = positive control). (B, hematoxylin & eosin, original magnification × 100; B (inset), original magnification × 400; C, avidin-biotin-complex immunoperoxidase, original magnification × 200).

A 54 year-old man with a previous diagnosis of lymphomatoid granulomatosis (LYG) presented with a left conjunctival growth consisting of clusters of papillae with focal hemorrhages of several weeks' duration (Figure 1A), during which time the systemic disease was thought to be in remission. The lesion was subsequently biopsied (Figure 1B). Given the patient's clinical history and the dense infiltrate of pleomorphic lymphoid cells in the tissue, immunohistochemistry was performed using antibodies against B-cell (CD20; Figure 1C), T-cell (CD45R0), and macrophage (CD68) markers. B-cell monoclonality was demonstrated using primer pairs FR3A, FR2A, and CDR3 for immunoglobulin heavy chain (IgH) regions [3], while PCR showed the presence of Epstein-Barr virus (EBV) DNA in the cells comprising the inflammatory infiltrate (Figure 1D). Based on the predominance of B- and T-cell infiltrates in the lesion, positive *IgH *rearrangement, and the presence of EBV DNA by PCR analysis, the patient's underlying disease was considered as a potential cause. He received palliative radiotherapy to the orbit.

### Case 2

**Figure 2:**
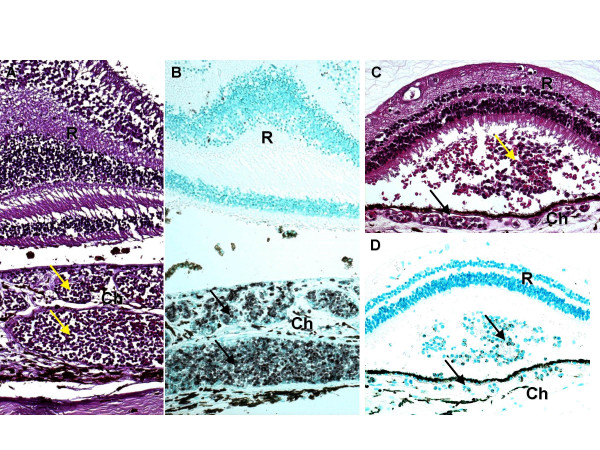
*Case 2*. (A and B) Choroidal vessels are filled with atypical cells (arrows), which are CD68+. (C) A subretinal infiltrate of leukemic cells (arrows), admixed with hemorrhage, is also seen. (D) The subretinal infiltrate and choroidal vessels contain a significant number of CD68+ cells (arrows). R = retina, Ch = choroid. (A and C, hematoxylin & eosin; B and D, avidin-biotin-complex immunoperoxidase; A and B, original magnification × 100; C and D, original magnification × 200).

A 51 year-old man with chronic myelomonocytic leukemia developed progressively worsening anemia, thrombocytopenia, and leukocytosis and subsequently expired. Post-mortem gross examination of the eyes was significant for multiple fresh and old retinal hemorrhages bilaterally. Microscopic examination revealed numerous atypical leukocytes within the choroidal vasculature (Figure 2A) and bilateral retinal hemorrhages, as well as a small focus of subretinal leukemic cells with admixed hemorrhage in the left eye (Figure 2C). The leukemic cells within the choroidal vasculature and in the subretinal lesion were strongly immunopositive for macrophage marker, CD68 (Figures 2B &2D). Molecular studies demonstrated negative IgH gene rearrangement and absence of EBV DNA.

## Discussion

The first case demonstrates a LYG metastatic lesion to the conjunctiva. First described as a distinct clinical entity in 1972, LYG is a rare, angiocentric, angiodestructive disease that is infrequently associated with ocular manifestations [4]. Though rare, the most commonly recognized pathology is consistent with the biopsy in this case, marked by the diffuse presence of B cells with an exuberant, attendant T-cell response. Current research suggests that LYG may be categorized as an EBV-related B-cell lymphoma [5]. This conjunctival lesion, confirmed immunohistochemically and molecularly, was the first clinical sign of LYG recurrence detected in this patient.

It should be noted that although ocular manifestations in LYG are uncommon, case reports do exist that document involvement of structures including the optic nerve [6], retina, sclera, and eyelid. Conjunctival involvement has also previously been described [7]. Reported clinical manifestations vary according to the ocular structure involved, but are generally diverse, ranging from ulcerative skin nodules [7] to sudden unilateral blindness to uveitis.

The second case involves a patient with chronic myelomonocytic leukemia (CMML), a disease formerly classified solely as a type of myelodysplastic syndrome (MDS) but reclassified in 1999 as a mixed MDS/myeloproliferative disorder [8].

Myelodysplastic syndrome (MDS) is a term that refers to a heterogeneous group of clonal bone marrow disorders associated with changes in marrow cellularity accompanied by dysmyelopoiesis and peripheral blood cytopenias [9]. Many case reports exist in the literature demonstrating ocular involvement in patients with MDS. In a 2005 retrospective study, Kezuka et al. reported that nearly half of 41 patients with MDS studied went on to develop ocular complications including corneal ulcer, iridocyclitis, vitreous hemorrhage, retinal hemorrhage, nerve-fiber layer infarcts, and optic neuritis [10].

In contrast to MDS, ocular involvement in patients with CMML is rarely reported, perhaps owing in part to the fact that the severe, progressive illness faced by many of these patients limits the feasibility of ocular examinations. While the patient described in the second case had no documented visual symptoms, pathology demonstrates infiltration of atypical cells within the vasculature of the choroid bilaterally as well as a subretinal hemorrhage with leukemic infiltration in the left eye. To our knowledge, this combination of findings in a patient with CMML has not been previously reported.

## Conclusion

We herein describe several microscopic ocular pathologic findings associated with two relatively rare hematologic malignancies. These cases emphasize both the ability of these diseases to demonstrate ocular involvement as well as important clinical and subclinical findings that may be seen with each. Furthermore, we emphasize the importance of an awareness of newly recognized manifestations of disease in cases such as these. In particular, the advent of novel antineoplastic therapies, with their associated potential to prolong life, may lead to the recognition of previously unobserved clinical signs. Our pathologic findings support that a routine eye examination, when possible, should be encouraged for patients with hematologic malignancies.

## Competing interests

The author(s) declare that they have no competing interests.

## Authors' contributions

JEH was involved in writing the manuscript and performing a review of the literature. DS performed the molecular analysis for both cases. MSM performed routine histology and immunohistochemistry for both cases. RJB was involved in the care of the patient in Case 1 and the review of the manuscript. CCC was involved in obtaining funding (NEI Intramural program) as well as conception of the report and critical review of the manuscript. All authors read and approved the final manuscript.

## Disclosure

JEH was a fellow in the 2006–07 Clinical Research Training Program, a public-private partnership supported jointly by the NIH and Pfizer, Inc. (via a grant to the Foundation for the NIH from Pfizer, Inc.).
